# Genital *Chlamydia trachomatis* infections in young adults – a school-based bio-behavioural study in urban areas, Poland, 2012 to 2015

**DOI:** 10.2807/1560-7917.ES.2018.23.6.17-00087

**Published:** 2018-02-08

**Authors:** Michal Czerwinski, Marta Niedzwiedzka-Stadnik, Anna Zielicka-Hardy, Anna Tomusiak, Malgorzata Sadkowska-Todys, Andrzej Zielinski, Magdalena Strus, Piotr Heczko, Magdalena Rosinska

**Affiliations:** 1 Department of Epidemiology, National Institute of Public Health – National Institute of Hygiene, Warsaw, Poland; 2 Department of Microbiology, Jagiellonian University Medical College, Krakow, Poland

**Keywords:** Chlamydia trachomatis, Prevalence, Young adults, School-based screening, sexually transmitted infection

## Abstract

One of the most common sexually transmitted pathogens disproportionately affecting young people is *Chlamydia trachomatis* (CT). This study aimed to assess prevalence of CT among sexually active students (aged 18–19 years) in their final years of high school education in Warsaw and Krakow. **Methods:** The sample was selected from 61 clusters, each cluster representing one school. We described city, sex, type of school and their association with CT prevalence. To account for non-responders we applied inverse probability weighting. **Results:** Our study population consisted of 3,136 young adults eligible for CT screening, of whom 2,326 reported having had sexual intercourse within past 12 months. Of the 950 students who agreed to be tested, 39 were infected with CT. Weighted prevalence of CT was 3.9% (95% confidence interval (CI): 2.7–5.1); however, prevalence in the students in Warsaw (6.6%; 95% CI: 3.5–12.4) was six times higher (prevalence ratio (PR) = 5.9; 95% CI: 2.0–17.3) than in Krakow (1.1%; 95% CI: 0.5–2.6). In both settings, female students attending vocational-technical schools were most affected; the prevalence in this group was more than five times higher (PR = 5.2; 95% CI: 1.7–15.6) compared with female peers in high schools and more than three times higher (PR = 3.3; 95% CI: 1.0–10.7) compared with male peers attending vocational-technical schools. **Conclusion*:*** Our study suggested prevalence of CT infection among young people in Poland comparable with the European average, supporting implementation of a CT control programme as recommended in international guidelines.

## Introduction

Infections caused by *Chlamydia trachomatis* (CT), with an estimated 131 million new cases worldwide each year, are among the most common sexually transmitted infections (STIs) [1]. Infection is often asymptomatic (75% in women and 50% in men), delaying treatment and increasing the risk of developing long-term reproductive sequelae, such as infertility. Untreated CT infections may also promote transmission of human immunodeficiency virus (HIV) [2].

Sexually active young people are disproportionately affected by CT infection. In the European Union (EU), two thirds of all reported CT cases occur in people younger than 25 years [3]. 

Early age of first sex, frequent change of partners and not using barrier contraceptive methods (condoms) are some of the identified drivers of CT transmission among young people [4]. The availability of effective and affordable treatment calls for the development of targeted screening strategies to identify and stop the spread of the infection and avoid long-term health effects. In Poland, no national strategy for STI control or a specific CT screening programme are currently in place. Elements of STI/HIV control are incorporated by schools into general health education sessions, on a voluntarily basis.

Despite the clear vulnerability of adolescents and young adults to CT*,* there are no comprehensive studies assessing this problem in Poland. Moreover, the majority of studies conducted to date have largely been based on adult patients actively seeking STI testing and/or treatment or visiting infertility clinics; consequently, they do not provide full insight into the prevalence of CT, especially among adolescents in the general population [5,6].

Our aim was to assess the prevalence of CT infection among sexually active young adults (aged 18–19 years) in their final years of high school education living in urban areas in Poland and to identify subgroups at particular risk of infection in order to target interventions accordingly.

## Methods

### Ethics statement

The survey was designed for students 18 years and older based on their self-reported year of birth. Young people under the age of 18 years were not included in this study as, in Poland, this requires parental consent which may have decreased the likelihood of student participation. All research participants provided written consent. To ensure participants remained anonymous, each person was assigned a unique identification number which they could use to obtain their results. Those with a CT-positive test result were offered confidential advice and medical treatment at one of the collaborating STI clinics.

All study procedures were approved by the Institutional Review Board of the National Institute of Public Health – National Institute of Hygiene (NIPH-NIH), Warsaw, Poland.

### Study outline

Between September 2012 and June 2015, a behavioural survey was conducted among students aged 18 years and older in their final years of secondary school education, in two of the biggest cities in Poland, Warsaw and Krakow. As ca 90% of 18-year-olds were enrolled in these schools, we did not sample young people who did not attend school.

The sample was selected using cluster sampling intending to include ca 60 schools (clusters) randomly drawn from all secondary schools registered in Warsaw and Krakow. Initially, we expected a 50% response rate and additional schools were sampled, to account for lower response rate. In each cluster (school), all students 18 years and older were given CT awareness lectures during which they were invited to participate in a self-administered behavioural survey*.* Awareness lectures were delivered by educators who routinely provide sexual health education for adolescents.

Subsequently, students eligible for CT screening (defined as those who had ever had a sexual partner, i.e. engaged in sexual intercourse or petting) were invited to provide urine samples for CT infection testing. The intended sample size for CT testing, assuming prevalence of infection was between 3% and 4.5%, ranged from 527 to 1,145 individuals.

### Behavioural survey

The main objective of the survey was to collect information on basic demographic data and risk factors associated with CT infection, including age at first sex, number and frequency of changing partners, condom use, symptoms of infection and previous history of STI infection or testing.

The questionnaire was designed by the Department of Epidemiology at the NIPH-NIH, using existing behavioural surveillance indicators and in consultation with sexual health educators. The questionnaire was pretested among 40 students in schools that were not selected to participate in this study, in order to assess the clarity of the questions and appropriateness of the language used.

### Urine sample collection for *Chlamydia trachomatis* screening

During awareness raising sessions, all students who had ever had a sexual partner were offered an opportunity to get tested for CT*.* They received a sterile urine collection container and were asked to provide a 10 mL urine sample. Students who wanted to be tested were asked to collect their first stream of urine (containing mucus and epithelial cells) and return the container to the chlamydia screening team. After centrifugation (3,000 rpm, 30 min), a pellet was collected and stored at -20 °C.

### Laboratory testing

Frozen urine specimens were transported to the Department of Microbiology at the Jagiellonian University Medical College in Krakow for CT testing by real-time nucleic acid amplification kit REALQUALITY RS-CHLAM T (AB Analitica, Padova, Italy) on an ABI 7500 real-time PCR system (Applied Biosystems, Foster City, California, United States).

### Statistical analysis

#### *Chlamydia trachomatis *prevalence

CT prevalence was calculated among all participants eligible for CT screening. To obtain an estimate of CT prevalence that accounted for non-responders, we fitted a two-step inverse probability weighting (IPW) regression model [7,8]. The main purpose of IPW was to correct for the fact that some subgroups of sexually active students (at varying risks of CT infection) were more willing to take part in the study and submit a urine specimen. Applying individual weights to each survey respondent who submitted a urine specimen allowed us to calculate the CT prevalence estimates unaffected by selection bias.

Using logistic regression, we compared characteristics of sexually active respondents who participated in CT screening with those that did not. We modelled the probability of urine specimen submission as explained by known predictors of CT infection. Variables were entered into the model in a stepwise fashion using only those factors with a p value <0.2 in the univariate analysis. 

In the next step, the inverse of the computed probabilities (after scaling) were applied as weights in multivariable log-binomial regression models used to estimate adjusted CT prevalence, prevalence ratios (PR) and their 95% confidence intervals (95% CI). 

Two log-binomial regression models were carried out: one including students who had ever had a sexual partner and the second comprising only students who were currently sexually active (i.e. reported having had sexual intercourse within the past 12 months). The final models included the following terms: city, age, sex, type of school and an interaction term between sex and type of school. The second model included one additional factor: type of partners (one steady partner vs multiple or casual).

We assumed that adolescents who attended the same school over a long period of time may have shared beliefs, attitudes and knowledge regarding sexual behaviours and STI prevention, which may influence their STI risk. To account for this within-school correlation, we used the generalised estimating method with an independent correlation structure commonly used to analyse clustered data with a binary outcome. We report weighted adjusted CT prevalence estimated from the final log-binomial model, assuming the distribution of the explanatory variables as in the total population.

#### Sexual behaviours

To better understand differences in CT prevalence, we compared patterns of partnership in the 12 months preceding the survey and other factors associated with STI risk (e.g. condom use, availability of STI prevention education programmes at school) among currently sexually active respondents of the behavioural survey, by subpopulation defined by sex and school type. Specifically, recent sexual behaviours (in the 12 months before the interview) among female students in vocational-technical schools were compared with three other major groups of students (female students in high schools; male students in high schools; male students in vocational-technical schools) using Tukey-type adjustment for multiple comparisons [9]. Analysis was performed using SAS 9.3.

## Results

### Selected schools

During the study period, 206 schools (140 high schools and 66 vocational-technical schools) were randomly selected from a total of 432 secondary schools registered in Warsaw and Krakow and invited to participate in the study. Of these 206 schools invited, 61 (30%) participated in the study; these were 38 schools officially classed as high schools and 23 vocational-technical schools. One of the participating high schools was privately owned.

Of the 145 schools that did not participate, 103 refused because of a timetable clash or the sensitive nature of our research topic. Forty-two schools were lost to follow-up or were unable to reach a final decision.

### Sample characteristics and predictors of participation in *Chlamydia trachomatis* screening

**Figure 1:**
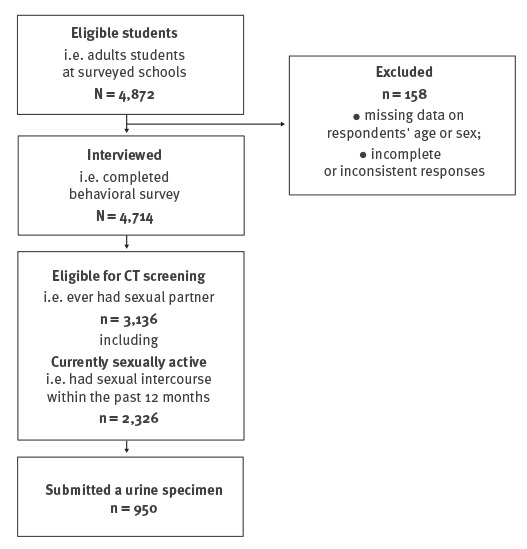
Inclusion process, school-based bio-behavioural study on genital *Chlamydia trachomatis* infections, Poland, 2012–2015 (n = 4,714)

Our study population comprised 4,714 young people aged 18 years and older of whom 2,768 were women and 1,946 were men (Figure 1). The mean age of respondents was 18.7 years.

Overall, 60% (n = 2,831) of respondents were attending high schools and 40% (n = 1,883) vocational-technical schools, however there were noticeable differences between the two cities (Table 1).

**Table 1:** Characteristics of the study population, school-based bio-behavioural study on genital *Chlamydia trachomatis* infections, Poland, 2012–2015 (n = 4,714)

Population by school type	Stratum	Krakow		Warsaw		p value^a^	Total	
		n	%	n	%		n	%
**Total study population (all interviewed students)**								
All schools	All	2,204	100.0	2,510	100.0	NA	4,714	100.0
	Women	1,356	61.5	1,412	56.3	0.0002	2,768	58.7
	Men	848	38.5	1,098	43.7		1,946	41.3
	18 years	698	31.7	1,239	49.4	<.0001	1,937	41.1
	19 years	1,160	52.6	1,043	41.6		2,203	46.7
	20 years and older	346	15.7	228	9.1		574	12.2
High school only	Women	496	22.5	1,259	50.2	<.0001	1,755	37.2
	Men	283	12.8	793	31.6		1,076	22.8
Vocational school only	Women	860	39.0	153	6.1		1,013	21.5
	Men	565	25.6	305	12.1		870	18.5
**Eligible for *Chlamydia trachomatis* screening (students, who had sexual intercourse or petting)**								
All schools	All	1,511	100.0	1,625	100.0	NA	3,136	100.0
	Women	933	61.7	884	54.4	<.0001	1,817	57.9
	Men	578	38.3	741	45.6		1,319	42.1
	18 years	449	29.7	722	44.4	<.0001	1,171	37.3
	19 years	795	52.6	717	44.1		1,512	48.2
	20 years and older	267	17.7	186	11.5		453	14.5
High school only	Women	267	17.7	764	47.0	<.0001	1,031	32.9
	Men	185	12.2	498	30.6		683	21.8
Vocational school only	Women	666	44.1	120	7.4		786	25.0
	Men	393	26.0	243	15.0		636	20.3

NA: not applicable.

**^a^** Chi-squared tests of the difference in proportions.

In total, 3,136 students reported having had a sexual partner in the past and were eligible for CT screening. Of these, 3% (n = 97) reported ever having been tested for any STI in the past. Of the total 4,714 students who completed the questionnaire, 55% (n = 2,613) reported having had sexual intercourse at least once, including 2,326 students who were currently sexually active (i.e. reported having had sexual intercourse within the past 12 months). This percentage was higher at vocational-technical schools (67%; n = 1,260) compared with high schools (48%; n = 1,353). Most respondents who had ever engaged in sexual intercourse were also sexually active in the previous 12 months (89%; n = 2,326).

**Figure 2:**
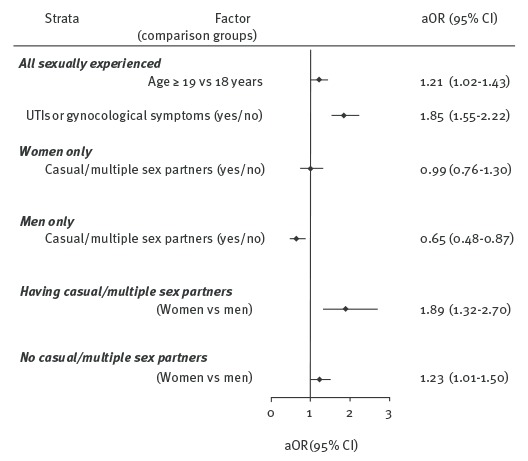
Factors influencing the submitting of a urine specimen, school-based bio-behavioural study on genital *Chlamydia trachomatis* infections, Poland, 2012–2015 (n = 3,136)

Of 3,136 students eligible for CT screening, 950 submitted urine specimen adequate for testing; 711 of them were currently sexually active, including 703 students who provided information on the number and type (steady or casual) of their partners in the 12 months preceding the survey. Overall, women were more likely to participate in CT screening than men, particularly those who had reported engaging in sex with casual or multiple partners in past 12 months (Figure 2).

aOR: adjusted odds ratio; CI: confidence interval; UTI: urinary tract infection.

In the multivariate analysis (logistic regression), among all students (female and male combined), the odds of screening increased with the respondents’ age (19 years and older vs 18 years) and current urogenital symptoms.

### *Chlamydia trachomatis* prevalence and related factors

Of the 950 students tested, 39 were infected with CT. The weighted prevalence of CT calculated for all respondents eligible for CT screening was 3.9% (95% CI: 2.7–5.1); however, there were considerable differences in CT prevalence estimates depending on the city. The inverse probability-weighted and adjusted (for age, sex and type of school) CT prevalence estimated in the final log-binomial regression model for Warsaw (6.6%; 95% CI: 3.5–12.4) was approximately six times higher (PR = 5.9; 95% CI: 2.0–17.3) than for Krakow (1.1%; 95% CI: 0.5–2.6).

The most affected socio-demographic group in both settings were female students attending vocational-technical schools (Table 2). Weighted CT prevalence in this group (after adjustment for city and age), was more than five times higher (PR = 5.2; 95% CI: 1.7–15.6) than among female peers in high schools and more than three times higher (PR = 3.3; 95% CI: 1.0–10.7) than among male peers attending vocational-technical schools. Among students attending high schools, however, similarly adjusted and weighted estimates of CT prevalence did not differ by sex.

**Table 2:** Overall and strata-specific inverse probability-weighted estimates of *Chlamydia trachomatis* prevalence among students, Poland, 2012–2015

Population by school type	Stratum	CT prevalence^a^		Tested	CT-positive
		%	95% CI	n	n
**Eligible for CT screening (students, who had sexual intercourse or petting ever; n=950)**					
All schools	Total	3.9	2.7–5.1	950	39
	Krakow	1.1	0.5–2.6	452	11
	Warsaw	6.6	3.5–12.4	498	28
	Women in Krakow	1.4	0.6–3.0	348	9
	Men in Krakow	0.9	0.3–2.6	104	2
	Women in Warsaw	8.2	4.3–15.4	287	17
	Men in Warsaw	5.4	2.4–12.2	211	11
High school only	Women in Krakow	0.6	0.2–2.1	68	1
	Men in Krakow	0.9	0.3–2.9	45	1
	Women in Warsaw	3.6	1.8–7.1	254	9
	Men in Warsaw	5.1	2.2–11.9	133	6
Vocational school only	Women in Krakow	3.1	1.8–5.4	280	8
	Men in Krakow	1.0	0.3–3.2	59	1
	Women in Warsaw	18.6	7.0–49.3	33	8
	Men in Warsaw	5.6	1.8–17.9	78	5
**Currently sexually active only (students who had intercourse in past 12 months; n=703)**					
All schools	Women with a one steady partner	3.8	2.3–6.3	377	18
	Women with a casual or multiple partners	3.7	1.8–7.7	106	6
	Men with a one steady partner	1.7	0.7–4.2	139	6
	Men with a casual or multiple partners	1.7	0.6–5.0	81	2
High school only	Women with a one steady partner	1.6	0.7–3.9	157	6
	Women with a casual or multiple partners	1.6	0.6–3.8	73	3
	Men with a one steady partner	1.7	0.6–5.0	70	3
	Men with a casual or multiple partners	1.7	0.6–5.6	38	1
Vocational school only	Women with a one steady partner	9.0	5.3–15.1	220	12
	Women with a casual or multiple partners	8.8	3.7–21.0	33	3
	Men with a one steady partner	1.7	0.6–5.5	69	3
	Men with a casual or multiple partners	1.7	0.4–6.5	43	1

CT: *Chlamydia trachomatis*.

^a^ Inverse probability-weighted and adjusted CT prevalence estimated in the final log-binomial regression models. These models included the following terms: city, age, sex, type of school and an interaction term between sex and type of school. In addition, the second model (only students who were currently sexually active) included type of partner/s (one steady partner vs multiple or casual).

CT prevalence calculated for currently sexually active students who reported one steady sex partner in the last year, was not statistically different from the prevalence noted among participants who reported casual or multiple partners (PR = 1.0; 95% CI: 0.5–2.3) (Table 2). Similarly, we did not find any association between CT prevalence and known risk factors for this infection such as early age (by 16 years) of first sex (PR = 1.2; 95% CI: 0.5–2.5) or oral contraceptive use (PR = 0.8; 95% CI: 0.4–1.4).

### Sexual behaviours among currently sexually active students, by population group

Overall, 2,326 respondents reported having had sexual intercourse in the previous 12 months. The majority of currently sexually active students (76%; n = 1,775) reported only one sex partner within the past 12 months, women (82%; n = 1,159) more so than men (67%; n = 616) (Table 3). Among women, those attending vocational-technical schools differed significantly from the rest of the sample (i.e. from women in high schools, men in high schools and men in vocational-technical schools) with respect to the characteristics of their partners; they were more likely to report only one steady sexual partner in the previous 12 months and less likely to engage in casual sex.

**Table 3:** Characteristics of currently sexually active^a^ women in vocational/technical schools compared with other groups of currently sexually active students, Poland, 2012–2015 (n = 2,326)

School type	Factor	Women	Men	Chi-squared p value
		%	%	
** Types of sex partners in the past 12 months **				
High school	One partner only, either steady or casual	78.8 *	70.1 *	< 0.0001
Vocational school		86.7 (ref)	63.9 *	
High school	One steady partner only	76.7 *	61.6 *	< 0.0001
Vocational school		86.4 (ref)	57.1 *	
High school	One casual partner only	2.1 *	8.5 *	< 0.0001
Vocational school		0.3 (ref)	6.7 *	
High school	Multiple partners: only steady, only casual or both steady and casual	21.2 *	29.9 *	< 0.0001
Vocational school		13.3 (ref)	36.1 *	
High school	Only steady multiple partners	4.6	4.1	< 0.0001
Vocational school		4.6 (ref)	10.2 *	
High school	Only casual multiple partners	2.8 *	11.4 *	< 0.0001
Vocational school		0.8 (ref)	9.1 *	
High school	Multiple partners, both steady and casual	13.8 *	14.4 *	< 0.0001
Vocational school		7.9 (ref)	16.9 *	
** Contraceptives in the past 12 months **				
High school	Condoms	74.0	80.2 *	< 0.0001
Vocational school		69.9 (ref)	82.2 *	
High school	Oral (hormonal) contraceptives	30.9	23.9	0.0062
Vocational school		27.6 (ref)	22.6	
High school	Withdrawal (Coitus interruptus)	18.9	16.2 *	0.0202
Vocational school		23.6 (ref)	20.8	
High school	Natural methods	13.1	11.9	0.3314
Vocational school		10.0 (ref)	12.6	
** Other **				
High school	No STI education at school	47.2 *	55.7 *	< 0.0001
Vocational school		67.2 (ref)	69.7	
High school	Never used any sources of information on STI	24.9 *	34.3 *	< 0.0001
Vocational school		45.6 (ref)	53.3	

STI: sexually transmitted infection.

^a^ Overall, 2,326 students reported sexual intercourse in the past 12 months before the interview, including 759 and 647 young women attending high schools and vocational schools, respectively, and 458 and 462 young men attending high schools and vocational schools, respectively.

* Indicates statistical significance (p < 0.05 after Tukey's multiple comparison adjustment) compared with female students in vocational-technical schools.

Overall, 19% (n = 166) of currently sexually active men and 28% (n = 381) of the women reported never using a condom in the previous 12 months. Otherwise, men and women were similar in terms of contraceptive use. In addition, respondents with a steady partner more often used oral contraception (PR = 1.6; 95% CI: 1.2–2.1). Students using oral contraceptives, compared with those who did not, were almost three times less likely (PR = 2.9; 95% CI: 2.5–3.3) to use condoms in the 12 months before interview.

Students attending vocational-technical schools were less exposed to STI prevention messages, be it at school (PR = 1.4; 95% CI: 1.3–1.5) or through other sources such as parents, peers, Internet, radio, television and print media (PR = 1.7; 95% CI: 1.5–1.9).

## Discussion

To date, several population-based surveys focusing on CT in Western Europe have been published [10-12]. However, data from central-eastern and eastern Europe are limited [13]. Our study based on 4,714 young people attending secondary school was the first population-based CT bio-survey conducted in Poland to date. We evaluated prevalence of CT infection among sexually active students (18 years and older) in their final years of high school education (n = 3,136) in two of the biggest cities in Poland (Warsaw and Krakow), and identified high-prevalence subgroups who need sexual health intervention. Importantly, we collected extensive information on personal characteristics and STI risk factors from all study participants to control and adjust for non-participation in CT screening. 

The weighted estimates of CT prevalence among all respondents eligible for CT screening varied considerably by city. The prevalence in Warsaw (6.6%; 95% CI: 3.5–12.4), even after adjustment for age, sex and type of school, was ca six times higher than in Krakow (1.1%; 95% CI: 0.5–2.6). Although the reasons for such heterogeneity are unclear, given the same study design and similar participation rates in both settings, they are likely to represent real differences in the local epidemiological profile. In line with our results, sub-national CT prevalence estimates in other countries are also very heterogeneous even after controlling for variations in the study design. Recent sub-national estimates in high-income countries ranged from 0.6% to 10.7% among women and from 1.1% to 5.9% among men 25 years or younger [14].

In our sample, the socio-demographic group with the highest prevalence of CT both in Warsaw and in Krakow were female students attending vocational-technical schools. The prevalence estimates for this group were more than five times higher than for female peers in high-schools, and more than three times higher than for male peers attending vocational-technical schools. Recent research links pockets of higher STI incidence among adolescents to certain social determinants of health, in particular low socio-economic status and limited access to health promotion or healthcare [10,15]. These results may be relevant for our study population. The choice of secondary high school track (vocational vs general education) is indeed strongly related to family background [16,17]. Consequently, the average socio-economic status of students at vocational-technical schools’ is likely to be lower compared with those attending general education schools [18].

We hypothesise that the higher prevalence of CT observed in vocational-technical schools reflects socio-economic differences as well as access to certain health services. Indeed, our data confirm that students attending vocational-technical schools were less exposed to STI prevention messages, be it at school or through other sources (i.e. parents, peers, Internet, radio, television and print media). The high prevalence of CT infections in this subgroup was unexpected, as female students in our sample who attended vocational-technical schools reported more commonly than other groups that they were in stable relationships. These students were more likely to engage in sexual intercourse with only one steady partner and overall less likely to report partner change.

It is worth noting, that in our sample, CT prevalence among respondents who reported only one steady partner in the last year was not statistically different from the prevalence among participants who reported one recent casual or multiple sex partners. These findings differed from research conducted in other countries where, age aside, the number of sexual partners was the only consistent risk factor for CT infection [19,20]. We note, however, that the majority of our respondents reported only one sex partner in the past year and we were not able to stratify the analysis by other important factors, i.e. condom use or age at first sex. Having casual/multiple partners could have been confounded by more consistent use of condoms [20]. It is also possible that some respondents were at increased risk for infection because of the behaviours associated with having steady partner. In our study, a steady partner was a significant predictor of oral contraceptive use. Moreover, couples using hormonal contraceptives were much less likely than non-users to have used condoms in the 12 months before the interview. Previous research has suggested that users of hormonal contraceptives may be at increased risk of acquiring STIs, although such association has not been observed in population-based studies [21,22]. Reduced use of condoms is, however, consistently associated with increased risk of STIs. Our finding also suggests, in line with previous studies, that large number of adolescents are more concerned about preventing unwanted pregnancies than STIs [23,24].

The study of sexual behaviour patterns is complex and, as demonstrated in this study, the effect of any specific risks may be confounded by other factors, making it difficult to base screening on risk assessment. For this reason, other studies such as one recently conducted in England, have not found an association of CT positivity rate with any of sexual behaviour variables [25].

Our study could be used as a platform to debate the implementation of a national STI control programme in which CT screening would form a key component. Opportunistic screening targeting, in the first instance, young women would be of benefit. In Poland, the coverage of STI programmes in school settings is extremely low and a large proportion of our respondents highlighted that they had no access to further information and resources on STI. According to guidelines from the European Centre for Disease Prevention and Control (ECDC) [26], such interventions should as a minimum include education for young people and case management with contact tracing.

### Limitations

Finding from this study may be limited due to participation bias, as we were only able to include 30% of invited sexually active students. Furthermore, our sample did not include a sufficient number of students attending private secondary schools, which prevents any extrapolation of the results to this small segment of the population (ca 6% of adolescents attend private secondary schools in the two cities).

The acceptance rate in our study was comparable to those reported in some school-based chlamydia screening programmes [27] but lower than in others [28]. Although our prevalence estimates were adjusted (IPW) for relevant factors associated with participation in CT screening, we note that surveys with lower response rates tend to produce higher estimates [1]. This is because participants in STI screening programmes usually have stronger risk factors related to STI risk than non-participants. The adjustment in our study allowed correction for a possible impact of the measured factors (i.e. age, sex, casual/multiple sexual partners, current UTI or gynaecological symptoms). Although we assessed similar predictors as reported in the literature [29], we recognise that additional predictors of participation may exist which were not accounted for.

Recent testing for CT was not likely to impact participation as only 3% of the participating and non-participating groups reported any STI test in the past. We therefore do not expect a large bias, despite the low response rate.

In addition, we detected only 39 CT infections, which limited our ability to conduct further analysis on risk factors for CT infection. We therefore analysed baseline data collected to control and adjust for non-participation in CT screening rather than examining subtle differences in behavioural risks. As a result, some social and behavioural variables which might have provided us with insight into the nature of excess risk for CT infection among adolescents were not available. This included patterns of partnership (e.g. having partners from or outside the school or having much older sex partners) or the students’ socio-economic status.

## Conclusions

This study suggests a prevalence of CT infection among young adults attending school in large Polish cities that is comparable to the European average and advocates the development of a CT control programme as recommended in ECDC guidelines. Our findings support the implementation of the key elements of these guidelines, e.g. sexual education and opportunistic screening, targeting the most vulnerable and underserved groups.

Our data suggest that female students attending vocational-technical schools in Poland, despite being the smallest group in our study, are at increased risk of CT. Generally, we note that the adolescents attending vocational schools have less access to sexual education and would benefit from targeted interventions to facilitate early detection of undiagnosed infection. More importantly, our findings confirmed that having only one steady partner in the recent year does not eliminate the risk for CT infection. Therefore, it should be recommended that clinicians offer routine screening and an STI consultation and advice to sexually active young women irrespective of their reported partner history. Universal educational programmes for secondary school students should be implemented in order to increase awareness and promote good sexual health.

## References

[1] Newman L Rowley J Vander Hoorn S Wijesooriya NS Unemo M Low N Global estimates of the prevalence and incidence of four curable sexually transmitted infections in 2012 based on systematic review and global reporting. PLoS One. 2015;10(12):e0143304. 10.1371/journal.pone.0143304 26646541 PMC4672879

[2] Fleming DT Wasserheit JN From epidemiological synergy to public health policy and practice: the contribution of other sexually transmitted diseases to sexual transmission of HIV infection. Sex Transm Infect. 1999;75(1):3-17. 10.1136/sti.75.1.3 10448335 PMC1758168

[3] European Centre for Disease Prevention and Control (ECDC). Sexually transmitted infections in Europe 2013. Stockholm: ECDC; 2015. Available from: https://ecdc.europa.eu/sites/portal/files/media/en/publications/Publications/sexual-transmitted-infections-europe-surveillance-report-2013.pdf

[4] Eaton DK Kann L Kinchen S Shanklin S Flint KH Hawkins J Youth risk behavior surveillance - United States, 2011. MMWR Surveill Summ. 2012;61(4):1-162. 22673000

[5] Choroszy-Król I Murawski M Pawlik L Teryks-Wołyniec D Frej-Mądrzak M Incidence of chlamydial uterine cervix infections in south−west Poland in the period of 1996-2004. Adv Clin Exp Med. 2006;15(3):427-33.

[6] Frej-Mądrzak M Teryks-Wołyniec D Jama-Kmiecik A Sarowska J Choroszy-Król I Diagnosing Chlamydia trachomatis urinary tract infections--preliminary report. Adv Clin Exp Med. 2015;24(3):441-5. 10.17219/acem/43719 26467132

[7] Brick JM Unit nonresponse and weighting adjustments: a critical review. J Off Stat. 2013;29(3):329-53. 10.2478/jos-2013-0026

[8] Seaman SR White IR Review of inverse probability weighting for dealing with missing data. Stat Methods Med Res. 2013;22(3):278-95. 10.1177/0962280210395740 21220355

[9] Elliott AC, Reisch JS. Implementing a multiple comparison test for proportions in a 2xc crosstabulation in SAS. Dallas: SUGI (SAS Global Users Group) conference; 26-29 Mar 2016; San Francisco, US. Available from: http://www2.sas.com/proceedings/sugi31/204-31.pdf

[10] Haar K Bremer V Houareau C Meyer T Desai S Thamm M Risk factors for Chlamydia trachomatis infection in adolescents: results from a representative population-based survey in Germany, 2003-2006. Euro Surveill. 2013;18(34):20562. 10.2807/1560-7917.ES2013.18.34.20562 23987832

[11] van Bergen J Götz HM Richardus JH Hoebe CJ Broer J Coenen AJ Prevalence of urogenital Chlamydia trachomatis increases significantly with level of urbanisation and suggests targeted screening approaches: results from the first national population based study in the Netherlands. Sex Transm Infect. 2005;81(1):17-23. 10.1136/sti.2004.010173 15681716 PMC1763744

[12] Goulet V de Barbeyrac B Raherison S Prudhomme M Semaille C Warszawski J; Prevalence of Chlamydia trachomatis: results from the first national population-based survey in France. Sex Transm Infect. 2010;86(4):263-70. 10.1136/sti.2009.038752 20660590

[13] Uusküla A Kals M Denks K Nurm U Kasesalu L Dehovitz J The prevalence of chlamydial infection in Estonia: a population-based survey. Int J STD AIDS. 2008;19(7):455-8. 10.1258/ijsa.2008.007325 18574116 PMC2918246

[14] Redmond SM Alexander-Kisslig K Woodhall SC van den Broek IV van Bergen J Ward H Genital chlamydia prevalence in Europe and non-European high income countries: systematic review and meta-analysis. PLoS One. 2015;10(1):e0115753. 10.1371/journal.pone.0115753 25615574 PMC4304822

[15] Hardwick D Patychuk D Geographic mapping demonstrates the association between social inequality, teen births and STDs among youth. Can J Hum Sex. 1999;8(2):77-90.

[16] Dustmann C Parental background, secondary school track choice, and wages. Oxf Econ Pap. 2004;56(2):209-30. 10.1093/oep/gpf048

[17] Putkiewicz E, Zahorska M. Społeczne nierówności edukacyjne – studium sześciu gmin. [Social inequalities in education – a study of 6 municipalities]. Warsaw: Institute of Public Affairs; 2001. Polish.

[18] World Health Organization Regional Office for Europe (WHO/Europe). Social inequalities in health in Poland. Copenhagen: WHO/Europe; 2012. Available from: http://apps.who.int/iris/bitstream/10665/107306/1/E96720.pdf

[19] Skjeldestad FE Marsico MA Sings HL Nordbø SA Størvold G Incidence and risk factors for genital Chlamydia trachomatis infection: a 4-year prospective cohort study. Sex Transm Dis. 2009;36(5):273-9. 10.1097/OLQ.0b013e3181924386 19265733

[20] Sonnenberg P Clifton S Beddows S Field N Soldan K Tanton C Prevalence, risk factors, and uptake of interventions for sexually transmitted infections in Britain: findings from the National Surveys of Sexual Attitudes and Lifestyles (Natsal). Lancet. 2013;382(9907):1795-806. 10.1016/S0140-6736(13)61947-9 24286785 PMC3899025

[21] Mohllajee AP Curtis KM Martins SL Peterson HB Hormonal contraceptive use and risk of sexually transmitted infections: a systematic review. Contraception. 2006;73(2):154-65. 10.1016/j.contraception.2005.08.012 16413846

[22] Torrone E Papp J Weinstock H Centers for Disease Control and Prevention (CDC) Prevalence of Chlamydia trachomatis genital infection among persons aged 14-39 years--United States, 2007-2012. MMWR Morb Mortal Wkly Rep. 2014;63(38):834-8. 25254560 PMC4584673

[23] Abel G Brunton C Young people’s use of condoms and their perceived vulnerability to sexually transmitted infections. Aust N Z J Public Health. 2005;29(3):254-60. 10.1111/j.1467-842X.2005.tb00764.x 15991774

[24] Matteelli A Capelli M Sulis G Toninelli G Carvalho ACC Pecorelli S Prevalence of Chlamydia trachomatis and Neisseria gonorrhoeae infection in adolescents in Northern Italy: an observational school-based study. BMC Public Health. 2016;16(1):200. 10.1186/s12889-016-2839-x 26927226 PMC4772514

[25] Syred J Engler B Campbell L Baraitser P Sheringham J Exploration of gender differences of Chlamydia trachomatis infection amongst young people reveals limitations of using sexual histories to assess risk in high-prevalence areas. Int J STD AIDS. 2014;25(8):564-70. 10.1177/0956462413515451 24352135

[26] European Centre for Disease Prevention and Control (ECDC). Guidance on chlamydia control in Europe – 2015. Stockholm: ECDC; 2016. Available from: https://ecdc.europa.eu/sites/portal/files/media/en/publications/Publications/chlamydia-control-europe-guidance.pdf

[27] Božičević I Grgić I Židovec-Lepej S Čakalo JI Belak-Kovačević S Štulhofer A Urine-based testing for Chlamydia trachomatis among young adults in a population-based survey in Croatia: feasibility and prevalence. BMC Public Health. 2011;11(1):230. 10.1186/1471-2458-11-230 21489313 PMC3090348

[28] Matteelli A Capelli M Sulis G Toninelli G Carvalho ACC Pecorelli S Prevalence of Chlamydia trachomatis and Neisseria gonorrhoeae infection in adolescents in Northern Italy: an observational school-based study. BMC Public Health. 2016;16(1):200. 10.1186/s12889-016-2839-x 26927226 PMC4772514

[29] Gravningen K Simonsen GS Furberg AS Wilsgaard T Factors associated with Chlamydia trachomatis testing in a high school based screening and previously in clinical practice: a cross-sectional study in Norway. BMC Infect Dis. 2013;13(1):361. 10.1186/1471-2334-13-361 23915415 PMC3751625

